# Etiology of pulp necrosis as a predictor of success in regenerative endodontics: a case report and bioinformatic analysis

**DOI:** 10.3389/fdmed.2025.1664854

**Published:** 2025-11-06

**Authors:** Marcela Alcota, Alfredo Torres, Paulina Ledezma, Montserrat Mercado, Paulina Paredes, Fermín E. González

**Affiliations:** 1 Department of Conservative Dentistry, Faculty of Dentistry, University of Chile, Santiago, Chile; 2 Laboratory of Experimental Immunology & Cancer, Faculty of Dentistry, University of Chile, Santiago, Chile

**Keywords:** dental trauma, regenerative endodontics, immature permanent teeth, bioinformatics, TNF

## Abstract

Regenerative endodontics (RE) is a biologically based procedure designed to replace damaged dental structures, including dentin and root structures, and cells of the pulp-dentin complex. It is the first-line treatment for patients with immature permanent teeth (IPT) that have lost vitality due to dentoalveolar trauma (DAT), caries, or developmental anomalies such as *dens evaginatus* (DE). Beyond resolving clinical signs and symptoms, RE allows for continued root development, substantially improving the long-term prognosis of these cases. Here, we present a clinical case of a 12-year-old female patient who, as a result of dentoalveolar trauma, exhibits asymptomatic apical periodontitis of tooth 1.1 and chronic apical abscess of tooth 2.1 in IPT. Both teeth were treated with regenerative endodontics, achieving successful RE in tooth 2.1 and inducing apical closure with Biodentine in tooth 1.1. In addition, we conducted a bioinformatic analysis to identify key genes associated with the success of post-trauma RE, providing a more complete and mechanistic understanding of the factors determining treatment success. In conclusion, the success of RE procedures in IPT may be conditioned by the type of DAT suffered by the tooth. Moreover, incorporating bioinformatic analysis introduces an innovative approach to unravel the molecular mechanisms involved in post-trauma dental regeneration.

## 1 Introduction

Immature permanent teeth (IPT) with pulp necrosis exhibit arrested root development, characterized by thin dentinal walls and short root length, increasing their susceptibility to fracture. This scenario is widespread in dentoalveolar trauma (DAT), which also carries a high risk of recurrence (1). In such cases, conventional endodontic therapy is contraindicated. While apexification can resolve infection, it does not support further root development, leaving the tooth structurally compromised.

Current evidence supports regenerative endodontics (RE) as the preferred approach for IPT with necrosis, as it promotes continued root maturation, increases dentinal thickness and length, and sometimes restores pulp vitality (2, 3). RE is indicated in teeth with up to two-thirds root formation and an open apex. In cases with near-complete root development and an open apex, either RE or immediate apexification with bioceramics (e.g., MTA or Biodentine) and obturation may be appropriate. When post-and-core restorations are needed, RE is contraindicated, and apexification is recommended (2).

RE is based on the tissue engineering triad: (i) stem cells from the apical papilla, (ii) bioactive growth factors released from dentin via chelation, and (iii) biomimetic scaffolds, such as a blood clot or autologous platelet concentrates (APC), including platelet-rich plasma (PRP) and platelet-rich fibrin (PRF) (2, 3). The protocol involves infection control, canal irrigation with 17% EDTA to release dentin-derived growth factors, induction of apical bleeding to form an intracanalar clot, and sealing the canal with a bioceramic material (4, 5). Infection control is critical; regeneration cannot occur in infected tissues (6). A second appointment, 1–4 weeks later, includes scaffold induction and definitive sealing.

Importantly, the extent and chronicity of pulp necrosis are key predictors of regenerative outcomes. Favorable prognosis is often observed in cases of recent trauma-induced necrosis, where apical papilla stem cells (SCAP) and Hertwig's epithelial root sheath (HERS) remain viable, allowing continued root development. In contrast, long-standing necrosis, severe infection, or teeth with nearly closed apices reduce the probability of pulp revascularization and root maturation, shifting the primary goal of therapy toward periapical healing rather than structural reinforcement. Different clinical factors, such as open apices, shorter duration of necrosis, and minimal periapical pathology, increase the likelihood of successful tissue regeneration, while advanced necrosis or heavy bacterial burden are associated with higher failure rates (1). Although the etiology of necrosis (trauma, caries, anomalous development) does not significantly alter success rates in immature teeth treated with regenerative procedures, it remains biologically plausible that the timing and environment of necrosis influence SCAP viability and regenerative potential. Thus, regenerative endodontics should be considered a first-line treatment for immature necrotic teeth, provided that disinfection protocols preserve apical cellular components (3).

Multiple clinical studies and case reports demonstrate that, despite variations in protocols—including scaffold type, irrigation solutions, and intracanal medications (e.g., calcium hydroxide or antibiotic pastes)—RE reliably resolves clinical symptoms, promotes apical healing, and has the potential to enhance root development (7, 8). However, the molecular mechanisms driving pulp regeneration and root maturation remain poorly understood. While clinical and radiographic outcomes are well documented, few studies have investigated the biological pathways underlying these processes. Integrating bioinformatic enrichment analysis of curated medical databases offers a novel and promising approach to identifying key molecular mechanisms involved in RE. To date, this methodology has not been applied in endodontics.

We report a clinical case of a 12-year-old female patient who presented with asymptomatic apical periodontitis in tooth 1.1 and a chronic apical abscess in tooth 2.1 following dentoalveolar trauma that occurred three years ago. Both teeth were treated with RE, with successful healing observed in tooth 2.1. Tooth 1.1, however, required apexification after RE failure.

## 2 Case report

This case report has been written according to the Preferred Reporting Items for Case Reports in Endodontics (PRICE) 2020 guidelines (9, 10). A 12-year-old patient with no history of systemic disease presented to a university-based endodontic clinic with a sinus tract in the anterosuperior vestibular region. The patient reported a dentoalveolar trauma at age nine and had received emergency care at a public hospital, where the anterosuperior teeth were splinted for three weeks, and a control radiograph was taken to rule out fracture. Supplementary Figure S1 depicts the flowchart of the case report following the PRICE 2020 guidelines.

Three years after the traumatic event, the patient attended the University Dental Clinic with no available follow-up records, radiographs, or referral notes, which represented a diagnostic challenge by limiting the reconstruction of the traumatic history. In the remote anamnesis, she reported a bicycle accident with direct impact to the maxillary anterior teeth, resulting in enamel–dentin crown fractures of teeth 1.1 and 2.1, associated with dental mobility and bleeding. Emergency care had been provided at the Roberto del Río Hospital, where the teeth were splinted for three weeks.

**Figure 1:**
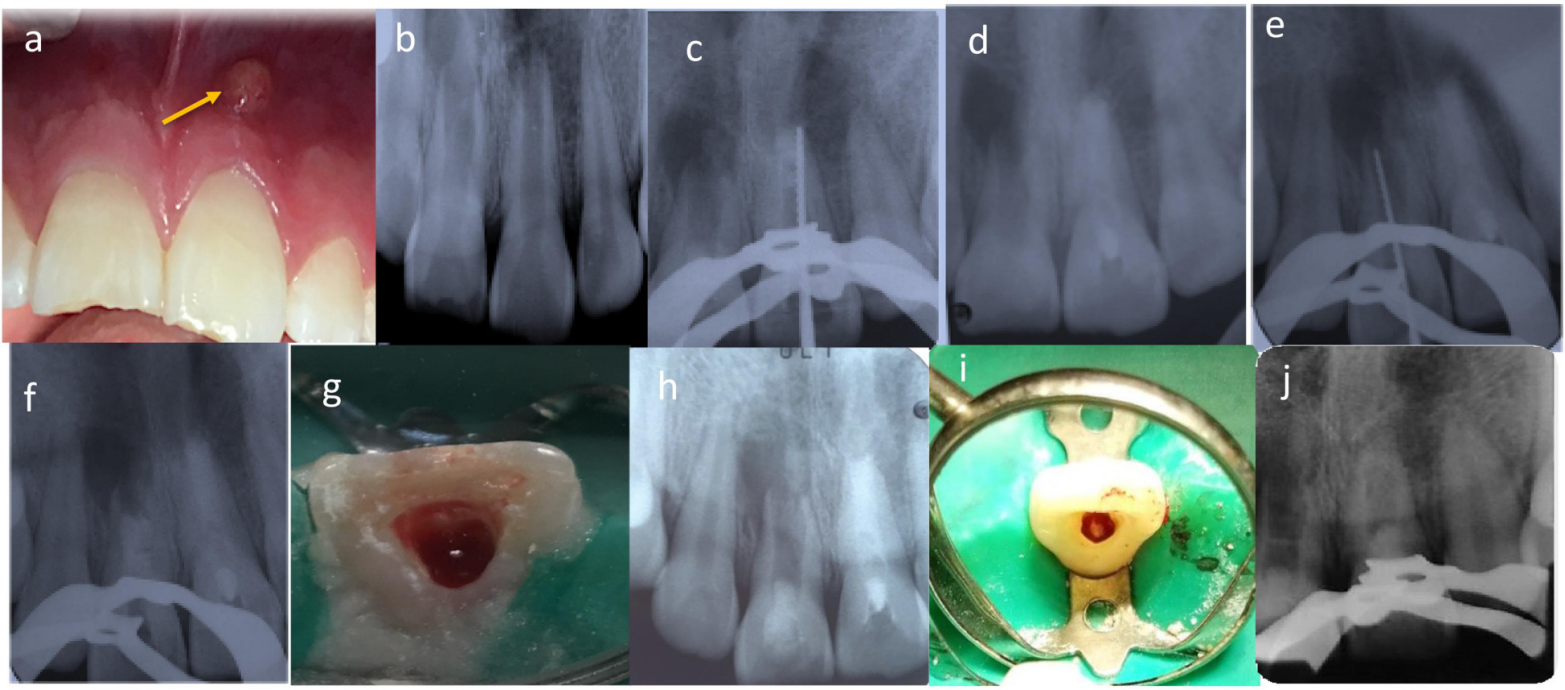
**(a)** Intraoral clinical examination revealed a vestibular parulis related to tooth 2.1. **(b)** Radiological assessment detected incomplete root formation of teeth 1.1 and 2.1. **(c,d)** tooth 2.1 first treatment session, determination of working length, instrumentation, and medication with CaOH (ultracal XS, Ultradent). **(e,f)** tooth 1.1 first treatment session, determination of working length, instrumentation, and medication with CaOH (ultracal XS, Ultradent). **(g)** RE of tooth 1.1. Apical bleeding was induced, forming a clot 3–4 mm below the CEJ. **(h)** Cervical area of tooth 1.1 was sealed with a bioceramic cement (Biodentine, Septodont), and the access was sealed with glass ionomer. **(i)** RE of tooth 2.1. The patient was asymptomatic, with a healed sinus tract. Apical bleeding was induced to form the blood clot. **(j)** Cervical area of tooth 2.1 was sealed with a bioceramic cement (Biodentine, Septodont), and the access was sealed with glass ionomer.

At the current examination, diagnostic methods included clinical inspection, pulp sensibility testing with cold stimulus, periapical radiographs, and cone beam CT. In addition, fistula tract tracing with a gutta-percha cone was performed in tooth 2.1, which presented a vestibular parulis. Clinical findings also revealed enamel–dentin fractures on teeth 1.1 and 2.1, with tooth 1.1 showing a slight vestibular displacement (Figure 1a). Both teeth were non-responsive to cold pulp testing. Radiographic evaluation, together with the fistula tract tracing in tooth 2.1, confirmed incomplete root formation and extensive periapical radiolucencies in both teeth (Figure 1b).

Based on the correlation of these findings, the differential diagnoses of pulp necrosis without periapical involvement and periodontal abscess were considered and ruled out. The final diagnoses were asymptomatic apical periodontitis for tooth 1.1 and chronic apical abscess for tooth 2.1, both affecting immature permanent teeth. The prognostic assessment highlighted that the immature root development and the extent of the periapical lesions negatively influenced the long-term prognosis. Regenerative endodontic procedures were therefore planned for both teeth.

The initial treatment session focused on tooth 2.1. Local infiltration anesthesia with 2% mepivacaine (Scandonest 2%, Septodont, France) was administered. The access cavity was prepared and abundantly irrigated with 1.5% sodium hypochlorite, and a radiographic length control with a N° 80K-file (Dentsply Maillefer, Ballaigues, Switzerland) was taken (Figure 1c). The working length was established by a second radiography taken 3 mm shorter than the apparent tooth length, and final irrigation was completed with saline solution. The canal was dried with sterile paper points No. 80 (Dentsply Maillefer, Ballaigues, Switzerland), and calcium hydroxide paste (UltraCal XS, Ultradent Products, Inc., South Jordan, UT, USA) was applied as intracanal medication. The access was sealed with a sterile cotton pellet, temporary cement (Fermin, Detax GmbH & Co. KG, Ettlingen, Germany), and glass ionomer cement (Chemfil, Dentsply Sirona, Charlotte, NC, USA) (Figure 1d).

The following day, treatment of tooth 1.1 commenced using the same protocol (Figures 1e,f). Both teeth remained medicated for one month.

**Figure 2:**
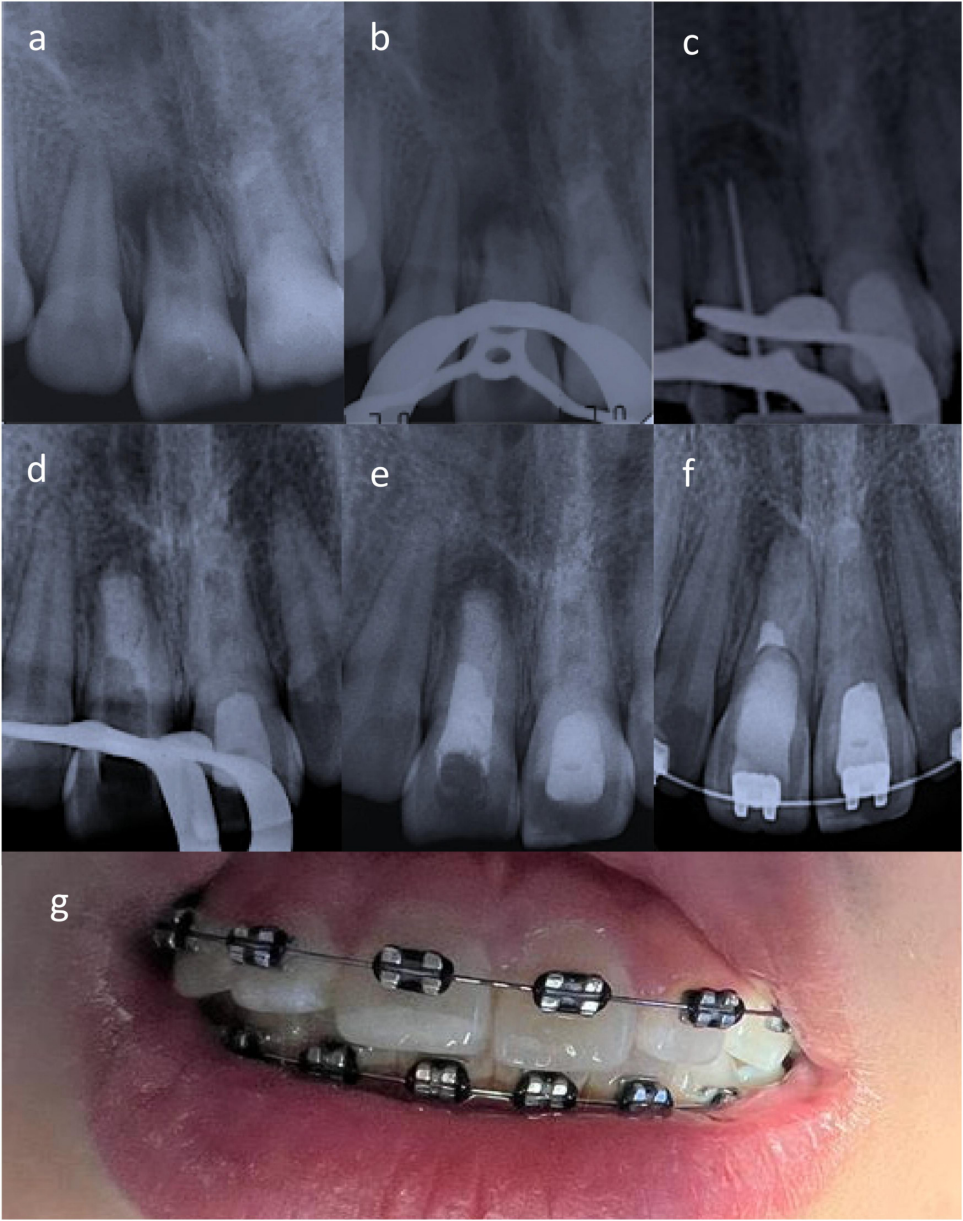
**(a)** Apexification induction of tooth 1.1 was indicated given the low likelihood of apical papilla cell presence due to the surgeon's curettage. **(b,c)** Upon removal intracanal medication suppuration was noted. **(d,e)** An apical plug with bioceramic cement and thermoplastic gutta-percha (apexification) was performed. **(f)** Radiographically, periapical repair was evident in teeth 1.1 and 2.1. **(g)** Clinically, the patient was in optimal conditions, with no clinical symptoms and restored teeth undergoing orthodontic treatment.

At the second appointment, one month later, the patient was asymptomatic (Figure 2a). Treatment continued with tooth 1.1, which was anesthetized with 3% mepivacaine (Scandicaine, Septodont, France). The temporary restoration and intracanal medication were removed with abundant irrigation using 1.5% sodium hypochlorite, followed by 3 ml of saline and 20 ml of 17% EDTA. After drying with sterile paper points, apical bleeding was induced with a No. 60K-file (Dentsply Maillefer, Ballaigues, Switzerland) to promote blood clot formation 3–4 mm below the CEJ (Figure 1g). The cervical third was sealed with bioceramic cement (Biodentine, Septodont, France), and the access was closed with glass ionomer cement (Chemfil, Dentsply Sirona, Charlotte, NC, USA) (Figure 1h).

One week later, the regenerative endodontic treatment of tooth 2.1 was completed. The patient remained asymptomatic, with no sinus tract or exudate in the root canal. Local anesthesia with 3% mepivacaine (Scandicaine, Septodont, France) was administered. The same protocol was followed (Figure 1i,j).

**Figure 3:**
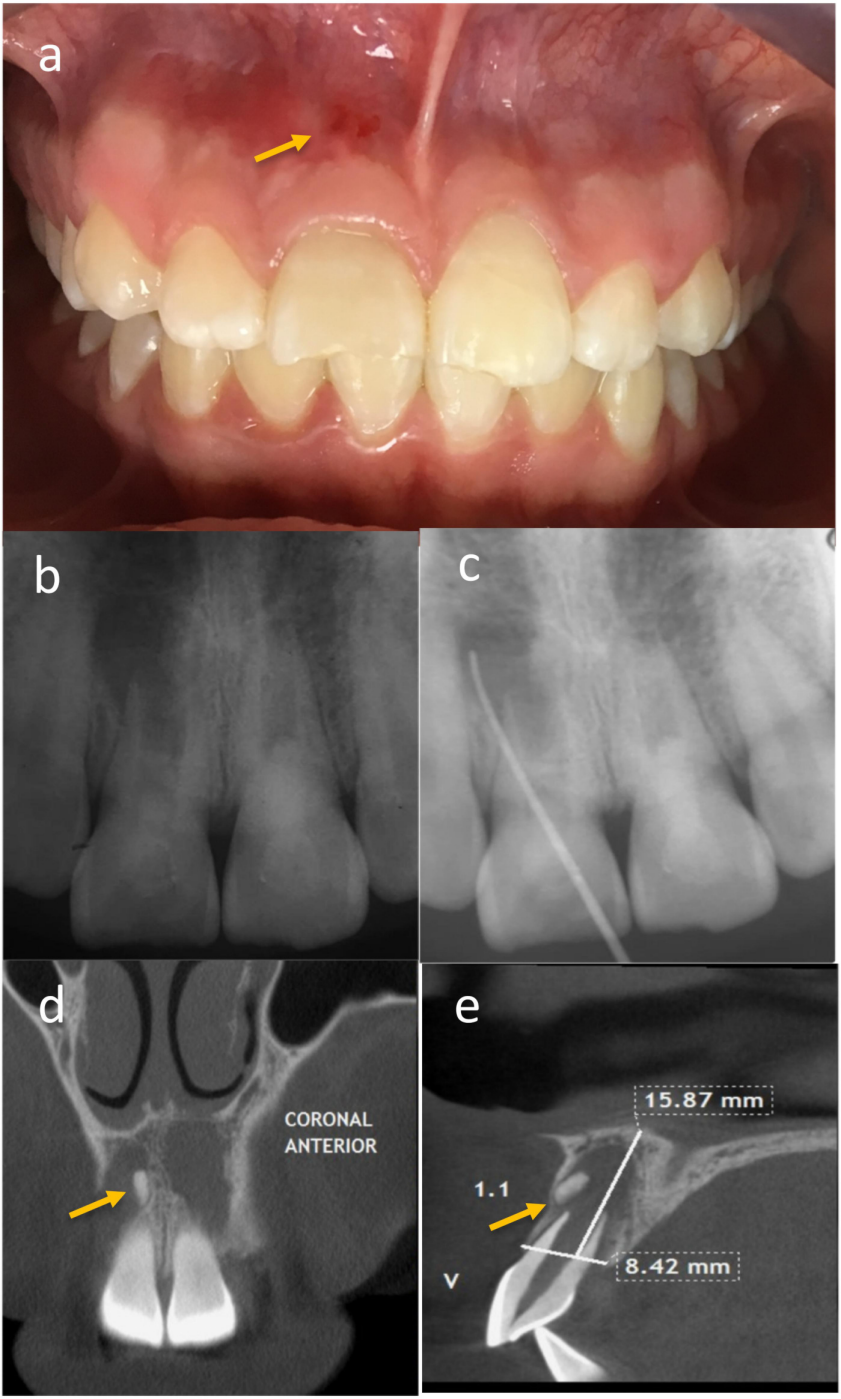
**(a)** Patient reported severe pain in tooth 1.1, and clinically, an erythematous area in the mucosa with pain on percussion and palpation of the vestibular fold was found. **(b)** A control radiograph was taken and the patient was maintained under observation. **(c)** An active sinus tract related to tooth 1.1 was observed, and fistula catheterization was performed. **(d,e)** A possible dental formation associated with tooth 1.1.

One month after both RE sessions, the patient returned to the scheduled control with severe pain in tooth 1.1 that had begun two weeks earlier. Clinical examination revealed an erythematous area in the mucosa of tooth 1.1, with pain to percussion and palpation of the vestibular fold (Figure 3a). A control radiograph was taken, and observation continued (Figure 3b). A composite restoration was placed on tooth 2.1.

At the subsequent follow-up, one month later, the patient was asymptomatic to percussion and palpation in both teeth. However, an active sinus tract related to tooth 1.1 was observed, so fistula catheterization was performed (Figure 3c), and a review of the initial Cone beam computed tomography (CBCT) detected a possible bone fragment associated with this tooth (Figures 3d,e). The patient remained under observation.

Another month later, the decision was made to refer the patient for surgical removal of the suspected bone remnant. An oral and maxillofacial surgeon performed the surgical procedure three months later. After the surgery, an acute apical abscess (stage 3) was reported, and subsequent controls showed the persistence of the sinus tract. Clinical examination revealed continued symptomatic percussion and palpation of the vestibular fold, with an active sinus tract related to tooth 1.1. The infection was controlled with 5.25% NaOCl irrigation and intracanal medication with calcium hydroxide paste (UltraCal XS, Ultradent Products, Inc., South Jordan, UT, USA). Given the likely absence of apical papilla cells due to the surgeon's curettage, apexification was initiated (Figure 2a).

Two months later, the patient was asymptomatic, and no sinus tract was observed; however, upon removing the provisional restoration and intracanal medication for tooth 1.1, intracanal suppuration was noted. The canal was abundantly irrigated with 5.25% NaOCl and medicated with calcium hydroxide and propylene glycol paste (Figures 2b,c).

A two-month control was postponed due to the COVID-19 pandemic. After 22 months, the patient returned with an active sinus tract and intracanal suppuration in tooth 1.1. The canal was disinfected with 5.25% NaOCl, and a bi-antibiotic paste (ciprofloxacin and metronidazole 1:1 in propylene glycol) was placed and maintained for three months.

The patient was asymptomatic three years after the initial visit, with no sinus tract or signs of infection (intracanal suppuration). An apical plug with bioceramic cement (Biodentine, Septodont, France) and thermoplastic gutta-percha (apexification) was completed (Figures 2d,e). Three weeks later, the definitive coronal restoration was placed.

Two months later, the patient remained asymptomatic. Clinical and radiographic examination revealed functional, asymptomatic teeth undergoing orthodontic treatment, with periapical healing evident in 1.1 and 2.1 (Figures 2f,g).

Finally, it is important to highlight that the patient and her caregiver were thoroughly informed during all treatment sessions regarding the clinical evolution and potential complications. Both demonstrated a high degree of responsibility and commitment throughout the therapeutic process, showing a strong interest in preserving the affected teeth.

## 3 Bioinformatic analysis

### 3.1 Identification of predictive coding-genes classification

Identifying and classifying predictive coding genes is a fundamental step in understanding complex biological processes and exploring disease mechanisms. The Génie datamining tool (http://cbdm-01.zdv.uni-mainz.de/∼jfontain/g_search_adv_wp.php) (11) was used to identify genes with strong predictive potential related to dental pulp regeneration. The query term “dental pulp regeneration after trauma” was used (accessed March 30, 2025). Inclusion criteria were restricted to studies with human data, while exclusion criteria filtered out orthologs and non-human datasets, resulting in 211 abstracts that comprised the training dataset (11). Since relevance in data mining methods is determined by the statistical prioritization of genes across the Medline corpus, to extract statistically significant coding genes, we applied a *p*-value cutoff of <0.01 for abstracts and a false discovery rate (FDR) <0.01 for gene selection. Fisher's exact test was employed to determine the strength of association between genes and the search topic.

### 3.2 Protein–protein interaction (PPI) network construction, functional enrichment, and cluster analysis

To explore the biological context of the identified genes, a functional enrichment analysis was performed using the ShinyGO v0.77 platform (http://bioinformatics.sdstate.edu/go/) (12). To contextualize the identified genes within their functional molecular relationships, a protein–protein interaction (PPI) network with a minimum confidence score of >0.4 was constructed using the STRING database (13) (http://www.string-db.org; accessed March 30, 2025), which then served as the foundation for subsequent clustering analyses (13).

K-means clustering groups features with similar expression patterns or annotations, enabling the identification of co-regulated genes, enriched pathways, and shared biological functions. The algorithm was applied to the PPI to categorize genes into functional modules, providing a foundation for downstream functional enrichment analyses. These clusters were then used to prioritize predictive coding genes, focusing on those with potential biomarker or therapeutic relevance.

### 3.3 Identification of hub genes in the key modules

Hub genes were defined as those with the highest intramodular connectivity within each functional cluster. To identify key hub genes, we evaluated the topological properties of nodes in the PPI network, considering features such as the number of connections, frequency of occurrence on shortest paths, closeness to all other nodes, and the influence of neighboring genes, among others. Using Cytoscape software (San Diego, CA, USA) (14), the cytoHubba plugin was applied to identify the top 10 hub genes per cluster. To maximize coverage while minimizing redundancy, seven nodal centrality algorithms were used: maximal clique centrality (MCC), maximum neighborhood component (MNC), degree, edge percolated component (EPC), closeness centrality, radiality, and stress (15). These metrics help pinpoint hub proteins that are critical for network function. To determine hub genes consistently ranked across multiple algorithms, we constructed an UpSet diagram, highlighting the most robust and biologically relevant hub candidates defined as core hub genes (14, 15). Functional characterization of these genes was conducted using the UniProt database (16).

## 4 Results

### 4.1 Identification of predictive coding genes associated with the query dental pulp regeneration after trauma

The gene dataset was identified in the Génie Datamining tool searching against articles in the whole Medline database matching the following PubMed query “dental pulp regeneration after trauma”. Two hundred eighty-five protein-coding genes were found as predictive datasets (Supplementary Table S1). This broad dataset reflects the genetic complexity underlying regenerative responses and provides the foundation for subsequent network and enrichment analyses.

### 4.2 Protein-protein interaction (PPI) network construction, biological functions, and molecular functions analyses

**Figure 4:**
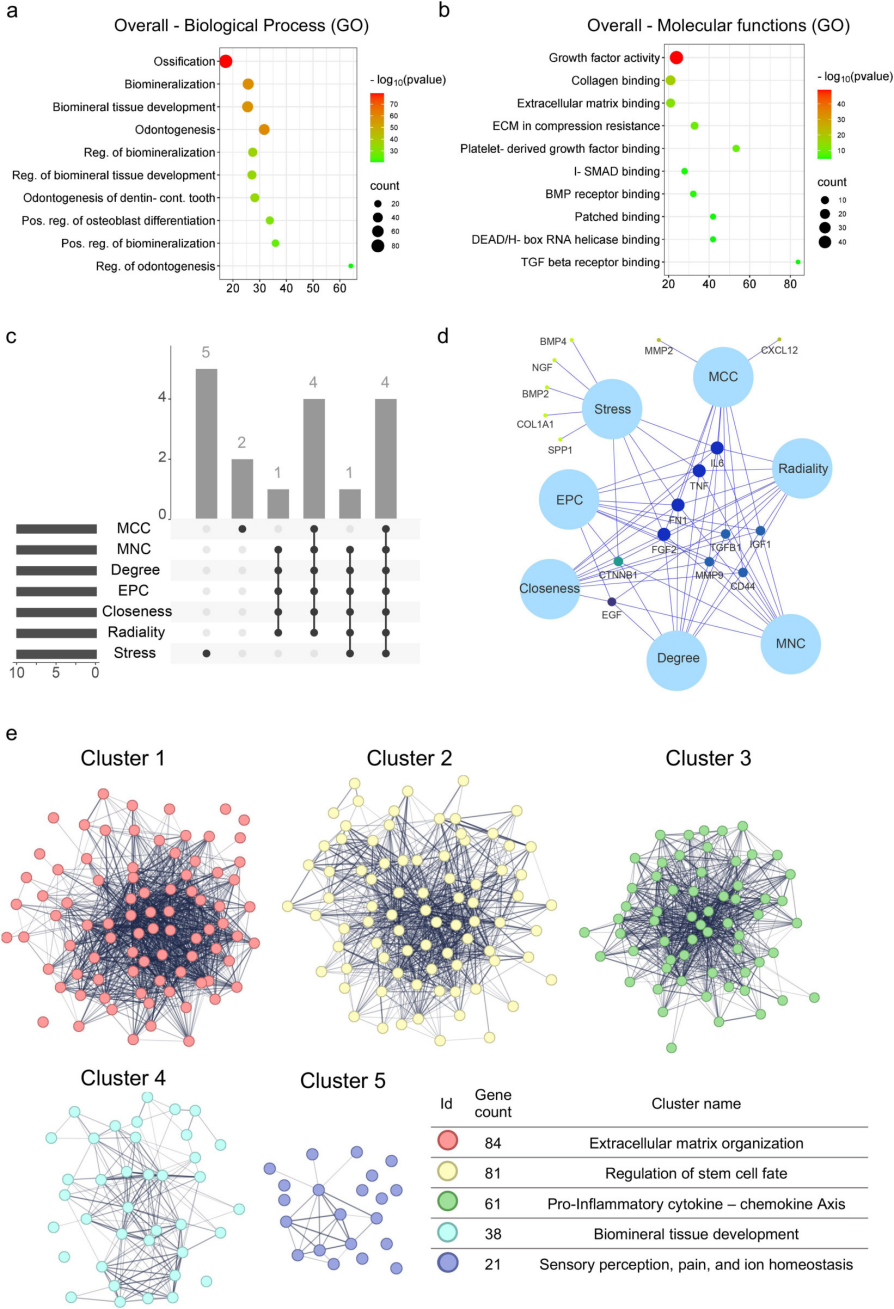
Enrichment analysis of predictive gene set associated with the “dental pulp regeneration after trauma” query. **(a)** Cluster module identification by K-means following protein-protein interaction (PPI) network construction. **(b)** An UpSet diagram shows that seven algorithms identified four overlapping hub genes. **(c)** Venn diagram displaying the four intersecting hub genes: FGF2, FN1, IL-6, and TNF. **(d,e)** Functional enrichment analysis of the predictive gene set, with annotations for enriched GO terms related to biological processes and molecular functions. An adjusted *p*-value < 0.05 was considered statistically significant.

The PPI network for the 285 protein-coding genes was constructed in STRING (Supplementary Figure S2). Gene Ontology (GO) Biological process and molecular function analyses were used in analyzing the PPI networks (Figures 4a,b). Based on GO enrichment, the biological process acted primarily on ossification, odontogenesis, biomineralization, biomineral tissue development, and regulation of biomineralization (Figure 4a). According to GO molecular function analysis, these genes were functionally related to growth factor activity, collagen binding, extracellular matrix binding, platelet-derived growth factor binding and I-SMAD binding (Figure 4b). These findings highlight that the identified gene network is strongly linked to tissue mineralization and extracellular matrix interactions, processes that are central to pulp healing and dentin formation after trauma.

### 4.3 Identification of hub genes

To further understand the functions and mechanisms of the identified proteins, the identification of hub genes that regulate dental pulp regeneration after trauma was performed (Supplementary Figure S2). An UpSet diagram was constructed to understand the relationships between sets and identify the overlapping hub gene for each method (Figure 4c). A Venn network was performed to visualize the four intersected hub genes (Figure 4d): FGF2, FN1, IL-6, and TNF. Finally, the detailed description and currently known functions of hub genes related to Dental pulp regeneration identified *in silico* are listed in Table 1. The identification of FGF2, FN1, IL-6, and TNF as common hub genes suggests that both growth factor signaling and inflammatory pathways are key regulators of pulp regenerative capacity, consistent with the dual requirement of tissue repair and controlled immune response.

**Table 1:** Description of hub genes.

UniProtKB identifier	Gene name	Description	Function	Cluster
P09038	FGF2	Fibroblast growth factor 2	Acts as a ligand for FGFR1-4 and integrins (e.g., ITGAV:ITGB3), essential for FGF2 signaling. Regulates cell survival, division, differentiation, and migration. Potent mitogen *in vitro*; induces angiogenesis.	Cluster 1
P02751	FN1	Fibronectin	Fibronectins bind cell surfaces and molecules like collagen, fibrin, heparin, DNA, and actin. They mediate adhesion, motility, opsonization, wound healing, and cell shape. Essential for osteoblast compaction, mineralization, and type I collagen deposition.	Cluster 1
P05231	IL-6	Interleukin-6	IL-6 is a cytokine involved in immunity, regeneration, and metabolism. It induces the acute phase response and supports host defense, though overproduction contributes to disease. Regulates Band T-cell differentiation, promotes TH17 generation, and acts on various cells including hepatocytes and CNS cells. Functions as a myokine and is essential for bone homeostasis and VEGF-mediated angiogenesis.	Cluster 3
P01375	TNF	Tumor necrosis factor	TNF is a cytokine secreted mainly by macrophages that binds TNFR1 and TNFR2. It induces tumor cell death, fever, cachexia, and can promote proliferation and differentiation. Impairs Treg function, stimulates VEGF (with IL-1β and IL-6) to drive angiogenesis, and promotes osteoclastogenesis and bone resorption.	Cluster 3

### 4.4 Functional enrichment analysis of modules identified by K-means clustering

Five significant modules of the PPI network were identified by K-means clustering (colored in Figure 4e). All modules integrated are illustrated in Supplementary Figure 41 and detailed in Supplementary Table S2.

Each cluster underwent functional enrichment analysis using the Gene Ontology database, revealing the following findings (Supplementary Figure S3): Cluster No. 1 was associated with “extracellular matrix organization” in biological processes (Supplementary Figure S3a). At the same time, molecular functions were enriched in terms related to “collagen, fibronectin, integrin binding, and extracellular matrix component binding” (Supplementary Figure S3b). Cluster No. 2 was associated with “Regulation of stem cell fate”, and the biological processes were primarily linked to “osteoblast differentiation” and “mesenchymal cell proliferation” (Supplementary Figure S3a). In contrast, molecular functions were related to “I-SMAD binding” and “patched and frizzled binding”, among others (Supplementary Figure S3b).

Cluster No. 3 showed a higher enrichment in terms of biological processes, “regulation of chemotaxis”, “regulation of leukocyte migration and proliferation”, and “acute-phase response” (Supplementary Figure S3a), while at the molecular functions, the most enriched terms were “C-X-C chemokine binding”, “interleukin-6 receptor binding”, and “chemoattractant activity” (Supplementary Figure S3b). On the other hand, Cluster No. 4 was mainly associated with biological processes related to “odontogenesis”, “biomineralization”, and “biomineral tissue development” (Supplementary Figure S3a). In contrast, molecular functions were dominated by “hydroxyapatite binding”, “alkaline phosphatase activity”, and “extracellular structural molecule activity” (Supplementary Figure S3b). Finally, Cluster No. 5 exhibited activities related to “sensory perception of pain and heat” in both biological processes and molecular functions, along with “transporter and ion channel activities” (Supplementary Figure S3a,b). Taken together, these clusters reveal that dental pulp regeneration after trauma is coordinated by modules governing extracellular matrix remodeling, stem cell fate, immune regulation, mineralization, and sensory pathways, underscoring the multifactorial nature of the regenerative process.

## 5 Discussion

Regenerative endodontics (RE) is a rapidly evolving field increasingly used to treat immature permanent teeth (IPT) with pulp necrosis (2, 3). As an emerging discipline, it lacks a universally accepted protocol, despite treatment guidelines published by the American Association of Endodontists (AAE) and the European Society of Endodontology (ESE) (17, 18). Current approaches involve multiple steps, materials, and variable concentrations, resulting in significant heterogeneity and numerous factors that can influence clinical outcomes (4, 18).

The primary objective of regenerative endodontics (RE) is the resolution of clinical signs and symptoms and radiographic evidence of apical healing, which is achieved in 91%–94% of cases (3, 8). In this clinical case, tooth 2.1 met this objective, while tooth 1.1 did not, due to a persistent sinus tract.

The secondary objective is continued root development, evidenced by apical closure, increased root length, and dentinal wall thickening (3, 8). Although RE has shown potential to promote these outcomes, they are not consistently predictable (19), with reported success rates ranging from 76% to 94% (8). Outcomes depend on factors such as the severity and duration of apical periodontitis, patient age, root development stage, trauma severity, and follow-up duration (3, 8). In this case, the secondary objective was fully achieved in tooth 2.1, but not in tooth 1.1, which eventually required immediate apexification using Biodentine and thermoplastic gutta-percha.

The tertiary objective is the recovery of pulp vitality, demonstrated by a positive response to vitality tests (3, 8). Histological studies have shown that the tissue formed is vascularized and innervated fibrous connective tissue, with cementoid/osteoid-like structures and nerve fibers, but lacking odontoblasts—hence not qualifying as proper pulp regeneration (20, 21). Moreover, few studies assess this parameter using reliable methods such as pulse oximetry or laser Doppler flowmetry, limiting its clinical relevance. In this case, vitality testing was not performed for tooth 2.1.

Several factors must be considered in the failure of RE treatment in tooth 1.1. The literature describes various intracanal medication protocols, with multiple studies supporting tri-antibiotic paste (TAP) as the most effective option (22, 23). Antibiotic concentrations between 0.3 and 1 mg/ml are recommended, as higher levels may impair apical stem cells (22, 23). However, the ESE advises against antibiotic pastes (18), while the AAE permits the use of Ca(OH)_2_, bi-antibiotic paste (BAP), or TAP (17). Both approaches have shown clinical success, though Ca(OH)_2_ is associated with enhanced SCAP proliferation and survival, whereas antibiotic pastes may reduce cell viability (4, 24). Moreover, antibiotic pastes can cause discoloration, cytotoxicity, allergic reactions, and antibiotic resistance (18, 24, 25). In this case, Ca(OH)_2_ was used in teeth 1.1 and 2.1. While tooth 2.1 responded successfully, tooth 1.1 showed persistent infection, intracanal suppuration, and a persistent fistula, despite prolonged treatment and vehicle variation. The infection resolved only after switching to BAP, suggesting a distinct microbial profile in tooth 1.1, potentially related to differing trauma types—an aspect discussed further below.

The Hertwig epithelial root sheath (HERS) plays a crucial role in root development by inducing differentiation of apical papilla stem cells into odontoblasts and of dental follicle/periodontal ligament stem cells into cementoblasts—key processes for the success of RE therapy (26). A recent systematic review reported a 60% failure rate of RE in IPT with necrosis due to trauma, such as avulsions or luxations (27). These injuries compromise the apical neurovascular bundle, reducing survival of key tissues like HERS and the apical papilla. In this case, RE failure in tooth 1.1 was attributed not only to persistent infection but also to trauma severity. Radiographic and photographic analysis revealed slight vestibular displacement of the tooth, and the patient's history included three weeks of splinting, suggestive of a combined injury involving an enamel-dentin fracture and lateral luxation. Such trauma likely impaired the apical structures, reducing the viability of stem cells essential for regeneration. Although this cannot be confirmed, as the emergency that occurred three years ago was not managed at our clinic, we hypothesize that the unfavorable outcome of regenerative endodontic therapy in tooth 1.1 may be attributed to the greater severity of the initial traumatic injury compared with tooth 2.1, considering that the latter, being its contralateral homologue, showed a more favorable evolution in the same patient.

At the molecular level, tissue repair and regeneration are orchestrated through complex cellular networks. Studying these interactions through systems biology and bioinformatics offers deeper insights (28). The clusters derived from bioinformatic analyses do not map directly onto categorical clinical steps; rather, they reflect complementary biological and molecular processes. Our in silico analysis identified cluster modules associated with tissue repair following trauma. Enrichment analysis highlighted the relevance of extracellular matrix (ECM) organization (cluster 1) and pro-inflammatory responses (cluster 3). Key hub genes—TNFα, IL-6, FN1, and FGF2—emerged as central mediators suggesting molecular pathways that may influence tissue healing. Importantly, these results do not provide direct evidence of expression in the present clinical case and should therefore be interpreted as hypothesis-generating, guiding future translational research.

The bioinformatic findings can be directly contextualized with the clinical outcomes observed in this case. For example, persistent inflammation and failure of regenerative endodontics in tooth 1.1 may reflect dysregulated activity of pro-inflammatory mediators such as TNFα and IL-6, which were identified as hub genes in our analysis. Inflammation plays a dual role in repair: it initiates healing by recruiting immune cells and promoting angiogenesis, but chronic inflammation can impair regeneration, drive fibrosis, and disrupt reparative signaling (29–33). Pro-inflammatory cytokines such as TNFα and IL-6, primarily secreted by M1 macrophages, are implicated in tissue repair and fibrotic processes (34–37). TNFα promotes fibroblast activation, collagen synthesis, and angiogenesis, while IL-6 regulates immune cell recruitment, angiogenesis, and progenitor cell proliferation (36, 37).

Conversely, the successful healing and continued root development in tooth 2.1 are consistent with reparative pathways involving FN1 and FGF2, both key regulators of extracellular matrix organization, angiogenesis, and tissue repair. These observations suggest that trauma severity may determine the balance between pro-regenerative and pro-fibrotic signaling, thereby influencing the likelihood of successful regenerative endodontic outcomes. Effective healing requires resolution of inflammation and transition to a pro-repair environment dominated by M2 macrophages. These cells release growth factors like VEGF and FGF2, supporting proliferation, angiogenesis, and ECM remodeling (38–40). M2 macrophages also stimulate fibroblasts to produce ECM components, including fibronectin (FN1)—a key protein in tissue scaffolding, cell migration, and immune cell recruitment (41, 42). Dysregulation of ECM assembly can lead to fibrosis, undermining regenerative outcomes.

In summary, both the nature of the traumatic injury and the host's inflammatory response critically influence the success of RE. Optimizing tissue healing requires not only control of infection and support for stem cell viability, but also precise regulation of inflammation and ECM dynamics.

To our knowledge, this is the first report combining a clinical case of regenerative endodontics after trauma with in silico bioinformatic analysis. This integrative approach is a strength, as it generates mechanistic hypotheses from curated datasets contextualized by clinical outcomes. However, limitations must be acknowledged: the single-case design prevents generalization; the analysis relied solely on the Génie platform, restricted to Medline English abstracts (11); no transcriptomic datasets of regenerative endodontics are available in public repositories for validation; and hub genes/modules were not experimentally validated. These factors underscore the exploratory nature of the work and the need for multi-center and experimental studies to confirm the proposed mechanisms.

## 6 Conclusions

RE is a promising treatment for immature permanent teeth with pulp necrosis and apical pathology, offering the potential for continued root development and improved long-term prognosis. By integrating clinical observation with in silico analysis, this exploratory study generates novel mechanistic hypotheses on how trauma severity may shape regenerative outcomes. Although limited by its single-case design and lack of experimental validation, it highlights the need for multi-center clinical research and molecular validation studies. Oral tissue repair is a complex process governed by immune responses and mediated by molecules such as FN1, FGF2, TNFα and IL-6, which were identified through our in silico analyses and are consistently reported in the literature as central regulators of regeneration. These mediators were not directly evaluated in the present case, but they provide important biological context for interpreting clinical outcomes. While acute inflammation promotes healing, persistent pro-inflammatory states can impair regeneration and favor fibrosis. A deeper understanding of these mechanisms is crucial for optimizing RE outcomes, and future regenerative strategies should aim to modulate the inflammatory microenvironment to enhance tissue repair, especially in trauma-compromised cases.

## Data Availability

The raw data supporting the conclusions of this article will be made available by the authors, without undue reservation.
