# Supplementary material for: Etiology of pulp necrosis as a predictor of success in regenerative endodontics: a case report and bioinformatic analysis
Source: Front Dent Med. 2025 Nov 6;6:1664854. doi: 10.3389/fdmed.2025.1664854 (PMC12631631; doi:10.3389/fdmed.2025.1664854)
Supplement: Supplementary file 1 [file Datasheet1.docx]

Figure S1

Figure S2

Figure S3

**FIGURE LEGENDS**

**Figure S1.** Timeline of events describing the flowchart of the case according to the PRICE 2020 case report guidelines.

**Figure S2.** Protein–protein interaction network construction and Hub genes identification. (a) Protein–protein interaction network construction. (b) Hub genes identified by different algorithms.

**Figure S3.** Cluster module profiles extracted by K-means in STRING and GO enrichment analysis of the modular genes. (a) Functional annotations and over-representation analysis of the top 10 most enriched biological processes. (b) Functional annotations and over-representation analysis of the top 10 most enriched Molecular functions. An adjusted p-value < 0.05 was considered statistically significant.
